# Synergistic effects of *Lactiplantibacillus plantarum* and *Acacia mangium* leaf tannin extract on the fermentation quality, digestibility, and metabolomic profile of *Indigofera* silage

**DOI:** 10.14202/vetworld.2025.3571-3593

**Published:** 2025-11-27

**Authors:** Farisha Rachma Azzahra, Irwan Susanto, Nahrowi Nahrowi, Rohmatussolihat Rohmatussolihat, Rusli Fidriyanto, Yantyati Widyastuti, Yulianri Rizki Yanza, Vincent Niderkorn, Roni Ridwan, Anuraga Jayanegara

**Affiliations:** 1 Study Program of Nutrition and Feed Science, Graduate School of IPB University, Bogor, Indonesia; 2 Department of Nutrition and Feed Technology, Faculty of Animal Science, IPB University, Bogor, Indonesia; 3 Center for Tropical Animal Studies, IPB University, Bogor, Indonesia; 4 Research Center for Applied Zoology, National Research and Innovation Agency (BRIN), Cibinong, Indonesia; 5 Research Center for Animals Husbandry, National Research and Innovation Agency (BRIN), Cibinong, Indonesia; 6 Department of Animal Nutrition and Feed Technology Faculty of Animal Science, Universitas Padjadjaran, Jatinangor Sumedang, Indonesia; 7 INRAE, VetAgro Sup, UMR Herbivores, Université Clermont Auvergne, Saint Genès-Champanelle, France

**Keywords:** *Acacia mangium*, digestibility, *Indigofera zollingeriana*, *Lactiplantibacillus plantarum*, metabolomics, silage fermentation, tannins

## Abstract

**Background and Aim:**

*Indigofera zollingeriana* is a high-protein tropical legume with potential as a sustainable ruminant feed; however, its ensiling is challenged by rapid proteolysis and ammonia accumulation. Incorporating lactic acid bacteria (LAB) inoculants and natural tannin sources may enhance fermentation quality and nitrogen preservation. This study evaluated the effects of *Lactiplantibacillus plantarum* inoculant and *Acacia mangium* leaf tannin extract, individually and in combination, on the chemical composition, ensiling characteristics, *in vitro* rumen fermentation, digestibility, and metabolomic profiles of *Indigofera* silage.

**Materials and Methods:**

A completely randomized design was used with four treatments: (R0) control, (R1) *L. plantarum* (10^6^ colony forming units/g FM), (R2) 1% *A. mangium* extract, and (R3) combination of both additives, each with five replicates. Silages were fermented anaerobically for 30 days. Analyses included proximate composition, pH, lactic acid, ammonia nitrogen (NH_3_-N), *in vitro* gas and methane (CH_4_) production, volatile fatty acids (VFA), digestibility, and untargeted metabolomics of both silage and rumen fluid using gas chromatography–mass spectrometry. Data were evaluated using a one-way analysis of variance, Duncan’s test, principal component analysis, and partial least squares discriminant analysis.

**Results:**

*L. plantarum* lowered (p < 0.05) silage pH and fiber fractions but increased NH_3_-N due to enhanced deamination. *A. mangium* tannins effectively suppressed proteolysis, reducing NH_3_-N by 11.85%. Their combination improved (p < 0.05) dry- and organic-matter digestibility (↑ ≈ 9%), increased the propionate proportion by 6.82%, and lowered the acetate-to-propionate ratio, indicating a shift toward more energy-efficient rumen fermentation without significant methane (CH_4_) inhibition. Metabolomic profiling identified 23 key metabolites in silage and 11 in rumen fluid; acacia tannins increased fatty acyl compounds (+14.3%) while LAB enhanced prenol lipids, reflecting modified lipid and oxygen-derived pathways that improve nutrient utilization.

**Conclusion:**

The combination of *L. plantarum* and *A. mangium* leaf extract synergistically improved *Indigofera* silage quality, nutrient preservation, and digestibility while modulating beneficial metabolites associated with rumen fermentation efficiency. This integrated additive strategy represents a sustainable and locally adaptable approach for tropical ruminant feed production.

## INTRODUCTION

*Indigofera zollingeriana* is a tropical legume recognized as a promising high-quality forage for ruminant production. It exhibits strong adaptability to marginal agroecosystems, including acidic soils and low-fertility lands, making it well-suited to tropical environments. Nutritionally, *Indigofera* contains approximately 25–29% crude protein (CP) [1], supporting high intake and digestibility in ruminants [2]. However, forage availability in tropical regions, such as Indonesia is highly influenced by seasonal climatic fluctuations, particularly during the dry season, resulting in feed shortages. Consequently, effective forage conservation methods are required to maintain a consistent year-round feed supply. Among these, silage production, an anaerobic fermentation process driven by lactic acid bacteria (LAB), is a practical strategy for preserving forage quality and prolonging storage life.

Ensiling leguminous forages, such as *Indigofera*, present specific challenges due to their high-protein content, which is susceptible to rapid microbial degradation during fermentation [3]. This process often leads to excessive proteolysis and deamination, resulting in ammonia accumulation and inefficient nitrogen utilization by ruminants [4]. The use of LAB inoculants, particularly *Lactiplantibacillus plantarum*, can enhance fermentation by accelerating pH decline through lactic acid production [5]. However, *L. plantarum* alone is insufficient to prevent proteolysis completely, necessitating complementary additives that protect proteins from microbial breakdown during ensiling.

Tannins, as naturally occurring polyphenolic compounds, have shown potential as silage additives because of their ability to form stable tannin–protein complexes resistant to microbial degradation. *Acacia mangium*, a leguminous tree extensively cultivated in Indonesia, represents an abundant and underutilized local tannin source, especially from leaf residues generated by the timber industry. Tannins from other botanical sources, including chestnut, quebracho, and gallnut, have been shown to reduce proteolysis and improve silage fermentation stability [6–8].

Despite increasing interest in improving the fermentation quality of tropical legume silages, a comprehensive understanding of the biochemical interactions between LAB and plant-derived tannins remains limited. Most existing studies have been restricted to temperate forages, such as alfalfa or clover, using imported or commercial tannin extracts (e.g., chestnut, quebracho, or gallnut), which differ substantially in structure and reactivity from tropical condensed tannins. Furthermore, the majority of these investigations have focused solely on fermentation end-products or proximate composition, without exploring the metabolomic mechanisms underlying additive–substrate interactions.

Information on the local adaptation and efficacy of *A. mangium* leaf tannins, a sustainable and abundant by-product of Indonesian forestry, remains scarce, particularly in combination with *L. plantarum*. The potential synergism between microbial acidification and phenolic protein-protection mechanisms has not yet been quantified for *I. zollingeriana* silage. Likewise, no study has simultaneously evaluated the fermentation characteristics, *in vitro* rumen fermentation profile, digestibility, and metabolomic fingerprints derived from this integrated additive system. The absence of such multidimensional data hinders the rational formulation of bio-based silage additives suited to tropical feed resources and limits understanding of how these interventions influence nitrogen metabolism and energy partitioning during rumen fermentation.

This study aimed to evaluate the individual and combined effects of *L. plantarum* inoculant and *A. mangium* leaf tannin extract on the chemical composition, fermentation dynamics, digestibility, and metabolomic profiles of *I. zollingeriana* silage. Specifically, the objectives were to:

1. Assess changes in silage quality indicators, including pH, lactic acid, ammonia nitrogen (NH_3_-N), and nutrient fractions, following additive application.
2. Determine the effects of the treatments on *in vitro* rumen fermentation characteristics, gas and CH_4_ production, volatile fatty acid (VFA) distribution, and nutrient digestibility.
3. Characterize the silage and rumen fluid metabolomic signatures using gas chromatography–mass spectrometry (GC–MS) to identify differential metabolic pathways associated with additive treatments.
4. Elucidate potential synergistic mechanisms between microbial inoculation and plant tannins in enhancing fermentation efficiency, nitrogen preservation, and ruminal energy metabolism.

Through this integrative approach, the study seeks to establish a scientific basis for the development of locally adaptable, eco-friendly silage additive strategies that improve feed efficiency, reduce nutrient losses, and support sustainable ruminant production under tropical conditions.

## MATERIALS AND METHODS

### Ethical approval

The animal handling procedures in this study, specifically the collection of rumen fluid from donor cattle, were conducted under the supervision of a licensed veterinarian and in accordance with the Animal Welfare Standards of the National Research and Innovation Agency (BRIN), Indonesia. Ethical clearance was granted by the Animal Ethics Committee of BRIN under approval number 047/KE.02/SK/03/2024.

Rumen fluid was obtained from fistulated Ongole crossbred cattle maintained at the Research Center for Applied Zoology (BRIN) solely for *in vitro* fermentation analyses. No animals were sacrificed for this study, and all sampling procedures were performed in a manner that minimized stress and discomfort.

All experimental activities conformed to the Animal Research: Reporting of *In Vivo* Experiments (ARRIVE) 2.0 guidelines and the national regulations on the care and use of research animals (Law No. 18/2009 and Government Regulation No. 95/2012 on Livestock and Animal Health). The study was designed to ensure animal welfare, biosafety, and environmental sustainability in accordance with institutional and international ethical standards.

### Study period and location

The study was conducted from July 2024 to May 2025. The production of *Indigofera* silage was carried out at the Laboratory of Feed Science and Technology, Faculty of Animal Science, IPB University (Bogor, Indonesia). Extraction procedures, analyses of silage chemical composition and fermentation product, as well as *in vitro* rumen fermentability and metabolomic assessments, were performed at the Genomics and Environment Laboratory and the Veterinary and Zoological Laboratory, BRIN, Cibinong.

### Experimental design

This study employed a completely randomized design with four dietary additive treatments to produce *Indigofera* silage:

- R0: *Indigofera* silage without additives
- R1: *Indigofera* silage with LAB inoculant (*L. plantarum*)
- R2: *Indigofera* silage with 1% *Acacia* leaf extract
- R3: *Indigofera* silage with a combination of LAB inoculant and 1% *Acacia* leaf extract.

Each treatment was replicated 5 times, yielding 20 experimental units. The *in vitro* incubation process was performed with five replications per treatment, and the sample allocation followed the same design. The silage and rumen fluid metabolomic analyses were performed with three replicates per treatment. The 1% inclusion level of *A. mangium* extract was selected based on the previous study by Sadarman *et al*. [9], indicating that tannin concentrations below 2% of dry matter enhance silage quality without inhibiting fermentation.

### Preparation of feed additives

#### A. mangium leaf extract

Fresh *A. mangium* leaves were collected from the forest area of the IPB University campus in Dramaga, Bogor. A total of 5 kg of fresh leaves were extracted using the microwave-assisted extraction method in a microwave oven at 135 W for 3 min [10]. This method was selected for its higher efficiency and environmental friendliness than conventional solvent extraction. After cooling to room temperature, the suspension was filtered under vacuum using filter paper until a clear filtrate was obtained. The filtrate volume was then adjusted to 40 mL (0.1 g/mL) for phytochemical analysis and stored in tightly sealed dark glass bottles at 4°C until use. Subsequently, the extract was used as a silage additive at an application rate of 1% fresh matter. Information related to the bioactive compound is presented in Table 1.

**Table 1:** Bioactive compound contents in acacia leaf extract (mg/g DM).

Bioactive compound	Concentration
Total phenolic extracts (GAE)	48.20 ± 3.48
Total flavonoids (QE)	18.44 ± 6.80
Tannin (TAE)	306.73 ± 31.04

DM = Dry matter, GAE = Gallic acid equivalent, QE = Quercetin equivalent, TAE = Tannic acid equivalent.

#### L. plantarum inoculum

The LAB inoculum (*L. plantarum* isolate 1A-2, BTCC570) was obtained from the BRIN culture collection with an initial population of 10^8^ colony-forming units (CFU)/mL. Before use, the inoculum was diluted with sterile distilled water and evenly applied to the fresh material to achieve a final population of approximately 10^6^ CFU/g fresh matter.

### Ensilage procedure

*I. zollingeriana* forage in the vegetative phase (60 days after sprouting) was harvested from the Agrostology Experimental Station, Faculty of Animal Science, IPB University (6°55′ S, 106°72′ E; 183 m a.s.l.). The average temperature at the sampling location was 31°C, with a relative humidity of 89%. The forage moisture content before treatment was 73%. Young leaves and branches were chopped to approximately 3 cm in length using a forage chopper.

The ensiling procedure followed the method of Kondo *et al*. [11], with four treatments: (R0) without additive (control), (R1) with *L. plantarum* addition, (R2) with 1% *Acacia* leaf extract addition, and (R3) with a combination of *L. plantarum* and *Acacia* leaf extract. The forage and additives were thoroughly mixed until homogeneous and then packed into 300-mL vacuum plastic bags (300 mL capacity). The bags were tightly vacuum-sealed to ensure anaerobic conditions and stored at room temperature (25°C–27°C) for 30 d. The ambient temperature was monitored daily using a digital thermometer with variations of 2°C. Anaerobic conditions were visually confirmed by the absence of leakage or bulging during fermentation.

After fermentation for 30 days, the silage was opened and divided into two portions. A 10-g subsample was diluted with 90 mL of distilled water, homogenized, and filtered for pH, NH_3_-N, and metabolomic analyses. The remaining material was dried at 60°C for 48 h, ground to pass through a 1 mm sieve, and used for chemical composition analysis and *in vitro* fermentation testing.

### Analysis of chemical composition

The main chemical composition of the silage samples was determined, including dry matter (DM), CP, neutral detergent fiber (NDF), acid detergent fiber (ADF), and acid detergent-insoluble crude protein (ADICP). The analysis was conducted using the Buchi NIRFlex N500 spectrometer with the Fourier transform near-infrared (FT-NIR) method, according to the protocol developed by Parastiwi *et al*. [12], providing a rapid, nondestructive, and efficient analytical approach for silage composition evaluation.

The instrument was calibrated and validated by the Genomic and Environmental Laboratory of BRIN using reference samples that had been previously analyzed by Association of Official Analytical Chemists [13], Van Soest *et al*. [14], and Licitra *et al*. [15] standard wet-chemical methods. The calibration model was routinely verified in the laboratory to ensure the accuracy and reproducibility of the prediction results. Each sample was analyzed in duplicate to enhance accuracy and minimize potential measurement bias.

### Determination of silage quality

The supernatant obtained from silage extraction after the ensilage process was used to determine silage quality parameters, including pH, lactic acid concentration, and NH_3_-N concentration.

- The silage pH was measured using a digital pH meter (Jenway Model 2505) calibrated with standard buffer solutions at pH 4.0 and 7.0 before measurement.
- The lactic acid concentration was determined according to the method described by Borshchevskaya *et al*. [16]. Briefly, 50 μL of the silage extract was transferred to a test tube and mixed with 2 mL of a 0.2% FeCl_3_ solution. The mixture was incubated at room temperature for 15 s, and the absorbance was measured at 390 nm using a ultraviolet visible (UV–Vis) spectrophotometer. The lactic acid concentration was calculated from a calibration curve prepared with standard lactic acid solutions.
- The NH_3_-N concentration was determined following the procedure described by Souza *et al*. [17]. A 100 μL aliquot of silage extract was mixed with 1.5 mL of phenol reagent and 1.5 mL of NaOCl reagent, followed by incubation in a water bath at 39°C for 15 min. After incubation, the absorbance was measured at 630 nm using a UV–Vis spectrophotometer, and the NH_3_-N concentration was calculated from a calibration curve prepared using standard ammonium chloride solutions.

To ensure accuracy and reproducibility, all instruments were recalibrated before each analytical session.

### In vitro rumen fermentation

Fermentation was conducted following Theodorou *et al*. [18]. Dried and finely ground silage samples (particle size <1 mm) were used as fermentation substrates. A 0.5 g sample was placed into a 100 mL glass incubation bottle, followed by the addition of 50 mL of a mixture of fresh rumen fluid and McDougall buffer solution (1:2, v/v).

The rumen fluid was collected from fistulated Ongole crossbred cattle before morning feeding. The rumen fluid was filtered through four layers of gauze and maintained at 39°C in a pre-warmed thermostat. Each bottle was flushed with carbon dioxide gas for approximately 1 min to create anaerobic conditions before being tightly sealed with a rubber stopper and aluminum crimp. The bottles were incubated in a water bath at 39°C for 48 h.

### Gas and CH_4_ analysis

Cumulative gas production was measured at 2, 4, 6, 8, 10, 12, 24, and 48 h using a 50 mL gas syringe. The measured gas volume was corrected against the blank (without substrate) and expressed as the total gas volume (mL). Before each measurement session, the gas samples were analyzed for their composition using a multi gas detector (BH-4S, Henan Bosean Electronic Technology Co., Ltd., China). CH_4_ production was expressed as the volume of CH_4_ produced per gram of DOM (mL g^-1^ DOM).

### Analysis of liquid fermentation and digestibility

After incubation for 48 h, the fermentation mixture was centrifuged at 6000 rpm for 10 min. The supernatant was used for VFA analysis using GC–MS (Shimadzu QP2010 SE, Shimadzu, Japan), following the procedure of Sarwono *et al*. [19]. A 2-mL aliquot of the supernatant was mixed with 30 mg of 5-sulfosalicylic acid dihydrate and centrifuged again at 13,000 rpm for 10 min. The filtrate was then passed through a nylon syringe filter (25 mm, 0.45 μm), and 1 mL of the filtrate was transferred into a GC–MS vial. The injection volume was 1 μL using a MEGA-WAX MS column (0.25 mm × 30 m). The NH_3_-N concentration was determined using the method described by Souza *et al*. [17].

The solid residue was used for the second incubation with 50 mL of pepsin–HCl solution (39°C, 48 h), as described by Tilley and Terry [20]. After incubation, the residue was filtered under vacuum, dried in an oven at 105°C for 24 h, and then washed in a furnace at 600°C for 6 h. The *in vitro* dry matter digestibility (IVDMD) and *in vitro* organic matter digestibility (IVOMD) coefficients were calculated based on the differences between the initial and final DM and organic matter (OM) weights.

### Silage extraction for metabolomics

Fresh silage (10 g) was mixed with distilled water (90 mL) and homogenized using a grinder. The homogenate was centrifuged at 21,100 × *g* for 10 min at 4°C, and the resulting supernatant was used for volatile compound analysis by headspace GC–MS. A 2-mL aliquot of the liquid phase was transferred into a 20-mL headspace vial.

The analysis was performed using a GC–MS instrument (Shimadzu QP2020, Shimadzu Co.) equipped with an SH Rtx-5ms GC Capillary Column, Restek Corporation, USA (0.25 mm i.d., 0.25 μm film thickness, 30 m length). A 1700 μL volume of headspace gas (1700 L) was injected at an injector temperature of 230°C. The interface temperature was maintained at 280°C, and the ion source was maintained at 230°C. High-purity helium (99.9%) was used as the carrier gas at a constant flow rate of 2.5 mL/min.

The oven temperature program started at 40°C (held for 3 min), increased to 210°C at a rate of 4°C/min, and was held for 2 min. The solvent cutoff time was set to 3 min, with an ionization energy of 70 eV and a mass range of 35–250 m/z. The retention index was calculated using a series of n-alkane standards with carbon chain lengths ranging from C8 to C20. Before analysis, the GC–MS system was calibrated using standard gas mixtures, and blank vials were periodically analyzed to ensure baseline stability and avoid carryover between samples.

### Rumen fluid metabolic analysis

The volatile compound profile in the rumen fluid was analyzed using headspace GC–MS. The rumen fluid samples were centrifuged at 21,100 × *g* for 10 min at 4°C to separate the supernatant from the solid fraction. A 2-mL aliquot of the supernatant was transferred into a 20-mL headspace vial for analysis. The analysis was performed using a GC–MS instrument (QP2020, Shimadzu Co.) equipped with an SH Rxi-5Sil MS capillary column (0.25 mm i.d., 0.32 μm film thickness, 30 m length).

A volume of 1700 μL headspace gas was injected at an injector temperature of 230°C. The interface temperature was maintained at 280°C, and the ion source was maintained at 230°C. High-purity helium (99.9%) was used as the carrier gas at a constant flow rate of 2.5 mL/min. The oven temperature program started at 40°C and was held for 3 min, increased to 210°C at a rate of 4°C/min, and held for 2 min. The solvent cutoff time was set to 3 min, with an ionization energy of 70 eV and a mass range of 35–250 m/z. The retention index was calculated using C8–C20 n-alkane standards. Blank vials and three replicate samples were analyzed to ensure system stability and data reliability.

### Compound identification and chemometric analysis

The volatile compounds in the silage and rumen fluid were identified by comparing the obtained spectra with the library and retention index values of the National Institute of Standards and Technology (NIST 20; https://webbook.nist.gov/chemistry/). Metabolite annotation was performed using the Human Metabolome Database (HMDB; https://hmdb.ca/metabolites). Peak area data from untargeted metabolomic analysis were used as input variables for principal component analysis (PCA) and partial least squares discriminant analysis (PLS-DA) to distinguish and group treatments based on variations in metabolite profiles [21]. Chemometric analyses were performed using MetaboAnalyst 6.0 (www.metaboanalyst.ca). Metabolites with Variable Importance in Projection (VIP) values >1.0 were considered significant contributors in differentiating treatment groups [22].

### Statistical analysis

The data obtained were analyzed using analysis of variance with the assistance of Statistical Package for the Social Sciences software version 25 (IBM Corp., NY, USA) after confirming the assumptions of normality. When significant differences among treatments were detected (p < 0.05), Duncan’s multiple-range test was used to compare treatment means.

## RESULTS AND DISCUSSION

### Nutrient composition and fermentation characteristics of Indigofera silage

#### CP and fiber fractions

The CP content of *Indigofera* silage ranged from 27.32% to 30.31% on a dry-matter basis (Table 2). The addition of feed additives significantly (p < 0.05) increased CP, with R1 exhibiting the highest value. Fiber fractions also differed significantly among treatments, as feed additives generally reduced NDF and ADF content (p < 0.05). The lowest NDF was observed in R2, whereas the highest reduction in ADF was observed in R3. In addition, the ADICP content decreased significantly (p < 0.05) in all treatments, with R2 showing the lowest value.

**Table 2:** Nutrient composition of *Indigofera* silage treated with various additives (% dimethyl sulfate).

Treatment	Variables				

	DM	CP	NDF	ADF	ADICP
R0	92.63^c^	27.32^a^	21.98^d^	19.28^d^	9.96^c^
R1	91.92^a^	30.31^d^	19.98^c^	14.79^c^	5.92^b^
R2	92.56^b^	28.82^b^	17.12^a^	13.29^b^	3.11^a^
R3	92.34^b^	29.59^c^	19.02^b^	11.79^a^	3.61^a^
SEM	0.04	0.02	0.14	0.23	0.17
p-value	<0.05	<0.05	<0.05	<0.05	<0.05

Means with different superscripts within a column are significantly different (p < 0.05). ADF = Acid detergent fiber, ADICP = Acid detergent insoluble crude protein, CP = Crude protein, DM = Dry matter, NDF = Neutral detergent fiber. R0 = I*ndigofera* silage; R1 = I*ndigofera* silage + L*actiplantibacillus plantarum*; R2 = I*ndigofera* silage+1% acacia leaf extract; R3 = I*ndigofera* silage+L*actiplantibacillus plantarum* + 1% acacia leaf extract.

The high CP content of *Indigofera* silage indicates that this forage can be a high-quality nitrogen source for ruminant livestock. This value is higher than that reported by Jayanegara *et al*. [23], who reported a CP content of 26.20% DM. Ensiling is generally accompanied by the degradation of proteins into simpler fractions, such as peptides and amino acids, thereby increasing the water-soluble nitrogen fraction. The highest increase in CP content was found in the treatment with *L. plantarum* inoculant, indicating that this fermentative microorganism contributes to nitrogen preservation by enhancing microbial biomass synthesis during fermentation. This is in line with the findings of Chen *et al*. [24], who stated that LAB not only play a role in lowering pH but also contribute to microbial nitrogen that can enhance the final CP value of silage. In addition, during the final stage of ensiling, the pH reduction induced by *L. plantarum* inhibited proteolytic bacteria, thereby protecting CP from degradation. The higher protein content in inoculated silage is associated with an initial increase in LAB, which subsequently suppresses undesirable microorganisms in the final fermentation stage [25].

In addition to the *L. plantarum* inoculum, *Acacia* leaf-extract application increased the crude-protein content. This improvement is attributed to tannin compounds, which form tannin–protein complexes resistant to microbial proteolytic enzymes during ensiling [26]. By preserving protein, more nitrogen is made available to rumen microbes, supporting microbial growth and protein synthesis and improving the digestibility of DM and OM [27, 28]. Consequently, nitrogen-utilization efficiency and overall production performance in ruminants, including weight gain and milk yield, may be enhanced.

#### Effect of additives on NDF, ADF, and ADICP

NDF and ADF contents in *Indigofera* generally range from 27% to 31% and 25% to 28% of DM, respectively [20]. The results show that the ensiling process with the addition of various types of additives generally reduces NDF and ADF concentrations, reflecting an increase in feed digestibility potential. These findings are consistent with those of Li *et al*. [29] and Zhao *et al*. [30], who showed that inoculation with *L. plantarum* reduces fiber fractions during ensiling. This effect is attributed to the production of organic acids, especially lactic and acetic acids, which lower the pH and partially hydrolyze plant-cell-wall components, primarily hemicellulose, while lignin remains relatively resistant [31]. The addition of *Acacia*-leaf extract also contributes to NDF and ADF reduction, likely through tannin interactions with proteins and, to a lesser extent, fiber [32]. The decrease in fiber fractions reflects the combined action of microbial fermentation, endogenous plant enzymes, and organic acids produced during ensiling [33].

In this study, the ADICP content in the *Indigofera* silage was lower than that reported by Jayanegara *et al*. [34], which was 6.10%–9.40% of the DM. A lower ADICP value indicates a reduction in the formation of a protein fraction unavailable to livestock. The *Acacia*-leaf extract used in this study contained tannin, a polyphenolic compound that can form strong complex bonds with proteins and feed fibers. These tannin–protein complexes are partially reversible under rumen conditions during ensiling, allowing proteins to be released for microbial use. As a result, despite some degradation during fermentation, the remaining protein retains higher biological availability and can support improved nitrogen-utilization efficiency in livestock.

### Fermentation quality of Indigofera silage

#### Silage pH and lactic acid production

The pH of the *Indigofera* silage significantly differed among treatments (p < 0.05) in response to the addition of various feed additives (Table 3), ranging from 4.64 to 4.91. Additives generally lowered the silage pH, with the greatest reduction observed in R3. The feed-additive treatments also significantly affected lactic-acid production (p < 0.05), with R3 exhibiting the highest concentration. In contrast, R1 significantly increased the N-NH_3_ concentration in the silage (p < 0.05).

**Table 3:** Fermentation characteristics of silage treated with various additives.

Treatment	Variables		

	pH	Lactic acid (g/kg)	NH_3_-N (mM)
R0	4.91^c^	36.70^b^	2.11^b^
R1	4.81^b^	42.83^c^	3.30^c^
R2	4.86^bc^	31.82^a^	1.86^a^
R3	4.64^a^	67.62^d^	2.19^b^
SEM	0.01	0.67	0.03
p-value	<0.05	<0.05	<0.05

Means with different superscripts within a column are significantly different (p < 0.05). NH_3_-N = Ammonia nitrogen. R0 = I*ndigofera* silage, R1 = I*ndigofera* silage + L*actiplantibacillus plantarum*, R2 = I*ndigofera* silage+1% acacia leaf extract, R3 = I*ndigofera* silage + L*actiplantibacillus plantarum* + 1% acacia leaf extract.

The highest pH value was recorded in silage that did not receive additive treatment, indicating suboptimal fermentation. This result agrees with Sadarman *et al*. [35], who suggested that feed materials with high-protein content generally have high buffer capacity, making it more difficult for them to experience a decrease in pH during the ensilage process and potentially resulting in low-fermentation quality. Therefore, the application of appropriate additives is essential for the preservation of *Indigofera*-based silages. The pH range of *Indigofera* silage in this study is below the values reported by Jayanegara *et al*. [34] (pH 5.27–5.57), indicating better fermentation quality. Inoculation with *L. plantarum*, either alone or in combination with *Acacia*-leaf extract, significantly lowered the pH of the silage. This decrease was closely related to the metabolic activity of *L. plantarum* in lactic-acid production during fermentation. As a homofermentative lactic acid bacterium, *L. plantarum* efficiently converts water-soluble carbohydrates into lactic acid, resulting in acidic conditions, as indicated by its lower pH values [36].

#### Interaction between L. plantarum and tannin compounds

However, the administration of *Acacia*-leaf extract alone tended to reduce lactic-acid production. This effect is caused by the antibacterial activity of tannin compounds in the extract, which can inhibit the growth of epiphytic lactic acid-producing microorganisms [37]. The combination of *L. plantarum* inoculant and *Acacia*-leaf extract, however, resulted in significantly higher lactic-acid concentrations, indicating a synergistic effect between these two additives.

This synergistic effect is likely due to tannin-suppressing proteolytic microorganisms, which reduce protein degradation and silage buffering capacity, creating favorable conditions for *L. plantarum*-induced lactic-acid fermentation. In addition, *L. plantarum* tolerates phenolic compounds, including tannins, through the production of gallate decarboxylase, thereby enabling their degradation [38]. During the aerobic phase, silage with a high lactic-acid content may experience an increase in pH due to lactic-acid consumption by yeasts, promoting the growth of aerobic microorganisms such as molds [24, 39]. The significant reduction in pH observed in the combination treatment reflects more efficient fermentation and suggests the potential for improved silage stability, although further investigation is required for aerobic stability.

#### NH3-N concentration and protein protection

The increase in N-NH_3_ concentration in *Indigofera* silage after inoculation with *L. plantarum* differed from the report by Dong *et al*. [40], who stated that *L. plantarum* can suppress NH_3_-N accumulation by reducing proteolytic enzyme activity and microorganisms due to the decrease in pH during fermentation. The increase in NH_3_-N concentration observed in this study was attributed to the delayed decrease in pH and the absence of protein-protective compounds, which allowed continuous proteolysis and resulted in higher amounts of NH_3_-N [41].

The lowest NH_3_-N concentration was observed in the *Acacia*-leaf-extract treatments. This effect can be attributed to the presence of tannin compounds, which inhibit protein degradation through two main mechanisms: the suppression of protease enzyme activity from both plants and microorganisms, and the formation of tannin–protein complexes that are highly resistant to degradation by hydrolytic enzymes [34, 35]. The effectiveness of tannins in reducing NH_3_-N concentration in alfalfa silage has also been reported by Chen *et al*. [7] and Ding *et al*. [42] using condensed tannins from quebracho, hydrolyzable tannins from chestnut, and tannic acid.

Low NH_3_-N concentrations and decreased ADICP in *Acacia*-leaf-extract-supplemented silage indicate that tannin–protein complex formation effectively suppresses protein degradation. Tannins improve nitrogen conversion efficiency and silage quality by minimizing protein loss during fermentation, making ruminant feed more beneficial. This mechanism is particularly advantageous because some protected protein is gradually released in the rumen, increasing the availability of amino acids in the small intestine and supporting overall nitrogen-utilization efficiency.

### *In vitro* rumen fermentation profiles and digestibility of *Indigofera* silage

#### Rumen pH and ammonia concentration

**Figure 1:**
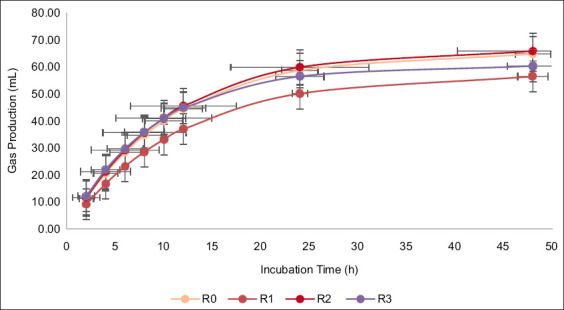
*In vitro* gas production (mL) of the treated *Indigofera* silage with various additives error bars represent standard deviations for each treatment. R0, *Indigofera* silage; R1, *Indigofera* silage + *Lactiplantibacillus plantarum*; R2, *Indigofera* silage + 1% acacia leaf extract; R3, *Indigofera* silage + *L. plantarum* + 1% acacia leaf extract.

The addition of different additives did not significantly affect the rumen pH of *Indigofera* silage, with values ranging from 6.83 to 6.85 (Table 4). In contrast, the concentration of NH_3_-N in the rumen fluid differed significantly (p < 0.05) among treatments, with R1 showing the highest NH_3_-N value. The IVDMD and IVOMD of *Indigofera* silage were also significantly influenced by additive supplementation (p < 0.05), with the greatest improvement observed in R3. After 48 h of incubation, the total gas production differed significantly among the treatments (p < 0.05), indicating a tendency for lower gas production in R1 (Figure 1). However, additive supplementation had no significant effect on CH_4_ production.

**Table 4:** *In vitro* rumen fermentation and digestibility of *Indigofera* silage treated with various additives.

Treatment	Variables					

	pH	NH_3_-N (mM)	IVDMD (%)	IVOMD (%)	Gas production at 48 h (mL)	CH_4_ (mL/g DOM)
R0	6.83	12.78^a^	59.97^a^	64.39^a^	64.88^b^	6.05
R1	6.83	28.32^c^	61.42^a^	66.70^ab^	56.52^a^	4.51
R2	6.85	25.60^bc^	61.82^a^	68.11^ab^	65.93^b^	6.02
R3	6.84	20.64^b^	65.64^b^	70.74^b^	60.40^b^	5.40
SEM	0.02	0.93	1.33	1.62	1.07	0.25
p-value	0.98	<0.05	<0.05	<0.05	<0.05	0.16

Means with different superscripts within a column are significantly different (p < 0.05). CH_4_ = Methane, IVDMD = I*n vitro* dry matter digestibility, IVOMD = *In vitro* organic matter digestibility, NH_3_-N = Ammonia nitrogen. R0 = I*ndigofera* silage, R1 = I*ndigofera* silage + L*actiplantibacillus plantarum*, R2 = I*ndigofera* silage + 1% acacia leaf extract, R3 = I*ndigofera* silage + L*actiplantibacillus plantarum*+ 1% acacia leaf extract.

The observed pH range remained within the normal physiological range (6.0–7.0), which supports the activity of fermentative microorganisms, particularly cellulolytic bacteria [43]. Furthermore, the use of buffers in the *in vitro* rumen fermentation system is intended to maintain pH stability, ensuring that environmental conditions remain optimal for rumen microbial activity during the incubation period [17]. *L. plantarum* inoculation did not significantly affect rumen pH [44]. Similarly, Huyen *et al*. [45] reported that the addition of tannin extract did not cause significant changes in rumen pH.

#### Rumen nitrogen dynamics

High levels of NH_3_-N in the rumen can indicate increased protein degradation, which poses a risk of significant nitrogen loss and negatively impacts physiological and reproductive performance [44]. One strategy for reducing protein degradation in the rumen is the formation of tannin–protein complexes, which aim to increase the bypass-protein fraction. However, the results of this study indicate that the low-dose addition of tannin extract was not effective in suppressing the rate of proteolysis in the rumen. This is due to the ability of rumen microorganisms to break down the tannin–protein complex; therefore, the protective effect on the protein is not optimal. This differs from Nasehi *et al*. [45], who reported that tannin can reduce plant protein degradation and increase amino acid bioavailability in the small intestine.

The variation in results can be explained by the type and concentration of tannins. Condensed tannin, the main tannin in *Acacia*-leaf extract, exhibits a lower ability to precipitate proteins than hydrolyzable tannins [46]. This property influences the strength of tannin interactions with rumen proteins and enzymes and determines biological activity [47]. Moreover, low concentrations of tannin (approximately 0.5%–3%) have not been reported to provide significant protective effects on protein in the rumen [37, 48]. The relatively short *in vitro* fermentation period may also limit tannin–protein interactions, thereby hindering the formation of stable complexes.

In a closed *in vitro* system, NH_3_-N cannot be absorbed through the rumen wall; its reduction depends on microbial assimilation. The low WSC content in *Indigofera* silage may further limit the available energy for microbial protein synthesis, thereby reducing nitrogen utilization efficiency. Forages with higher WSC contents improve the energy–nitrogen balance, enhancing microbial-nitrogen conversion and decreasing ammonia losses. Nevertheless, the NH_3_-N concentration observed remained within the optimal range to support microbial activity [49]. These findings suggest that combining *L. plantarum* and *Acacia* tannins enhances rumen fermentation efficiency and promotes nitrogen conservation, potentially reducing nitrogen losses to the environment.

#### Digestibility, gas, and CH_4_ production

These findings differ from previous studies by Huyen *et al*. [50] and Min *et al*. [51], which reported that tannin addition decreases *in vitro* digestibility. The observed increase in IVDMD indicates an associative effect between the two additives. Although tannin binds to fiber components [34], the observed increase in IVDMD suggests low affinity for fiber, allowing normal ruminal fermentation. *L. plantarum* accelerates pH decline and stabilizes fermentation, helping to reduce excessively strong tannin–protein complexes and allowing cellulolytic enzyme activity to remain optimal. Variations in tannin effects may result from differences in plant sources and chemical structures that influence biological activity [52]. Combining tannins with *L. plantarum* can mitigate the adverse effects of tannins by promoting a microbial community more adaptive to phenolic compounds [52]. These findings suggest that tannin effects on digestibility depend on type, dosage, and interaction with fermentative inoculants.

In an *in vitro* rumen incubation system, gas production results mainly from carbohydrate fermentation, with protein fermentation contributing less [53]. Lower gas production in *Indigofera* silage with *L. plantarum* addition may result from decreased fermentable-carbohydrate availability, as *L. plantarum* consumes soluble carbohydrates during ensiling, producing lactic acid [5, 54]. Consequently, limited substrates remain for rumen microbes during incubation.

Tannins in *Acacia* extract can modulate rumen fermentation by binding to structural carbohydrates, starch, and proteins, reducing substrate availability [7]. The magnitude of this effect depends on the type of tannin and the characteristics of the feed. Chestnut tannins did not affect gas production during *in vitro* incubation, consistent with the findings of this study [55]. Conversely, gallnut tannins combined with *L. plantarum* reduced fermentative activity [56], whereas high tannin doses suppressed rumen-fermentation efficiency [51]. These findings indicate that low tannin concentrations have minimal inhibitory effects on macronutrient availability.

#### CH_4_ emission and energy partitioning

*L. plantarum* inoculation tended to reduce CH_4_ production compared with other treatments. A previous *in vitro* study by Cao *et al*. [57] has reported that *L. plantarum* application did not affect IVDMD in vegetable silage but could reduce CH_4_ production. The variability in response depends on substrate type, buffering capacity, fermentation characteristics, and ensiling duration [58, 59]. CH_4_ reduction can occur through two mechanisms: direct inhibition of methanogen activity or indirectly through decreased carbohydrate degradation [60, 61]. In this study, *Acacia*-leaf-extract tannins did not significantly reduce CH_4_, likely due to a low tannin concentration that was insufficient to inhibit methanogens. Chestnut and Leucaena tannins reduce CH_4_ by 40% and 47%, respectively [62, 63]. Additionally, the *Acacia* extract used was unpurified and may contain non-tannin compounds that affect its activity [64].

### Rumen fermentation end-products

#### VFA profile

The total VFA concentration in the rumen fluid significantly differed among treatments (Table 5). No significant difference was observed between R0 and R2, whereas R1 and R3 showed a significant decrease (p < 0.05), with R3 exhibiting the lowest value. Acetic- and butyric-acid proportions were similar, whereas propionic-acid proportion increased significantly (p < 0.05) in R3. In contrast, R1 and R2 exhibited lower propionic acid relative to R0. The acetate-to-propionate ratio (C_2_:C_3_ ratio) increased in R1 but decreased in R3.

**Table 5:** Profile of VFAs in rumen fermentation with various additives *in vitro*.

Treatment	Total VFA (mM)	Acetic acid (% total)	Propionic acid (% total)	Butyric acid (% total)	C_2_:C_3_
R0	65.45^b^	66.22	19.65^bc^	7.82	3.37^a^
R1	54.99^ab^	69.72	17.33^a^	7.30	4.03^b^
R2	61.60^b^	65.85	19.16^b^	8.30	3.45^a^
R3	44.35^a^	65.05	20.99^c^	7.36	3.10^a^
SEM	2.01	0.58	0.21	0.25	0.07
p-value	<0.05	0.10	<0.05	0.50	<0.05

Means with different superscripts within a column are significantly different (p < 0.05). C_2_:C_3 =_ Acetate-to-propionate ratio; VFA = Volatile fatty acid. R0 = I*ndigofera* silage, R1 = I*ndigofera* silage+L*actiplantibacillus plantarum*, R2 = I*ndigofera* silage + 1% acacia leaf extract, R3 = I*ndigofera* silage + L*actiplantibacillus plantarum* + 1% acacia leaf extract.

VFA production reflects carbohydrate fermentation activity and is a major energy source for ruminants [65]. The total VFA concentration here was relatively low compared with the normal range (70–130 mM) [49], likely due to limited fermentable carbohydrate availability. The lowest total VFA was observed in R3, consistent with Hapsari *et al*. [41], who reported that *L. plantarum* inoculation can reduce VFA. Tannins may enhance this effect by forming complexes with proteins and carbohydrates, limiting available substrates [66].

Acetic and butyric acids are the main precursors for long-chain fatty acid synthesis, while propionic acid is the main substrate for gluconeogenesis, the key glucose source in dairy metabolism. *L. plantarum* inoculation increases acetic-acid concentration through organic-acid production during ensiling and modulates rumen fermentation, improving feed digestibility and animal performance [67, 68]. The addition of *Acacia* tannins did not significantly affect acetate proportion, differing from reports showing increased acetate following tannin supplementation [62, 63], possibly due to differences in tannin type or substrate.

The propionate proportion here was higher than that reported by Chen *et al*. [56]. The combination of *L. plantarum* and *Acacia*-leaf extract increased propionate content, suggesting improved energy availability. This increase may result from lactic acid accumulation during ensiling, which rumen microbes convert to propionate during fermentation [11]. Leguminous forages tend to produce higher propionate, explaining the nonsignificant changes in butyrate proportions [67, 69].

#### Energy efficiency and CH_4_ mitigation potential

The high C_2_:C_3_ ratio in *L. plantarum*-only treatment likely relates to lower gas and hydrogen (H_2_) production due to the limited availability of fermentable substrates. In contrast, combining *L. plantarum* and *Acacia* extract increased propionate concentration, possibly because tannins inhibited acetogenic bacteria, shifting H_2_ use toward the propionate-formation pathway. Propionate-producing bacteria preferentially utilize H_2_ as an electron donor [70]. The resulting decrease in the C_2_:C_3_ ratio indicates a shift toward a more energy-efficient fermentation pathway, which contributes to lower CH_4_ emissions and improved feed utilization. Although CH_4_ reduction was not statistically significant, the altered fermentation profile suggests that higher tannin inclusion could further enhance H_2_ use for propionate synthesis. The increased proportion of propionate reflects improved ruminal energy metabolism, supporting greater microbial protein synthesis and enhanced animal performance.

### Untargeted metabolome profiles of silage from *Indigofera*

#### Overview of volatile compound identification

**Figure 2:**
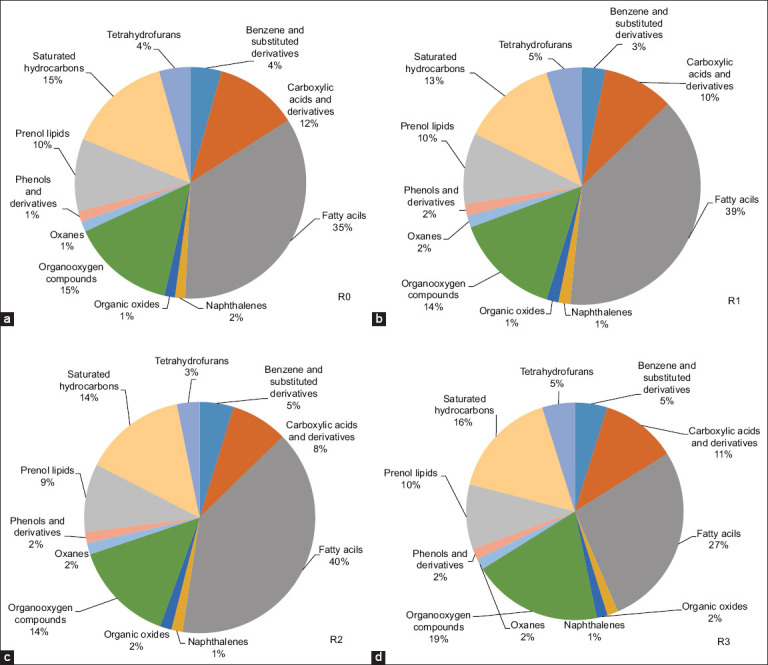
Class-based variation of metabolite compounds in *Indigofera* silage as classified by the Human Metabolome Database. Treatments included (a) R0, the control; (b) R1, Lactiplantibacillus *plantarum* inoculation; (c) R2, acacia leaf extract supplementation; (d) R3, combined application of both additives.

Untargeted metabolomic analysis of the *Indigofera* silage was conducted using GC–MS. This approach enabled the comprehensive characterization of metabolite profiles under different feed additive treatments. The results revealed clear variations in metabolite composition between the control and treatment groups. A total of 74 volatile compounds were successfully identified using the NIST 20 library based on their retention indices, retention times, and mass spectral matching. Subsequently, the identified compounds were classified into several metabolite classes according to the HMDB (Figure 2).

The detected metabolites were distributed across major classes, including saturated hydrocarbons, benzene and substituted derivatives (e.g., naphthalenes and phenols), carboxylic acids and derivatives, lipids (fatty acyls and prenol lipids), and organooxygen compounds (e.g., tetrahydrofurans, oxanes, and organic oxides). The proportion of fatty acyls increased significantly in R1 (39%) and R2 (40%) compared with R0 (35%), but decreased in R3 (27%). In addition to fatty acyls, saturated hydrocarbons (13%–16%) and organooxygen compounds (14%–19%) were consistently detected across all treatments.

### Chemical nature and functional role of detected compounds

Organic compounds encompass a diverse group of chemical species in which one or more carbon atoms are covalently bonded to other elements, primarily oxygen, H_2_, or nitrogen [71]. Carboxylic acids and their derivatives are key fermentation products that play an essential role in maintaining silage stability. Aromatic compounds, including benzene derivatives, naphthalenes, and phenols, were detected in relatively low proportions but remained significant due to the presence of phenolic compounds derived from the tannins in *Acacia* leaf extract, which exhibit antimicrobial and antioxidant activities.

Minor classes, such as tetrahydrofurans, oxane, organic oxides, and prenol lipids, further reflect the metabolic diversity generated during the fermentation process.

### Multivariate statistical analysis of silage metabolome

#### PCA and PLS-DA

**Figure 3:**
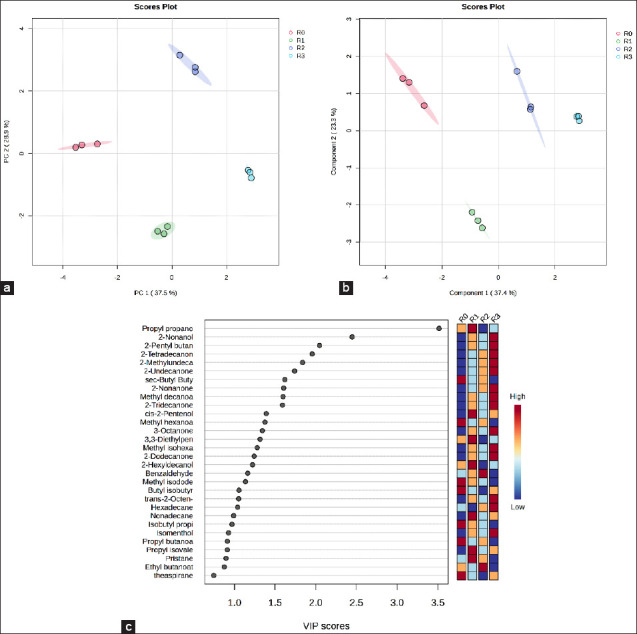
Metabolite profiles of *Indigofera* silage with different feed additives. (a) Principal component analysis score plot illustrating the variation among treatments. (b) Partial least squares discriminant analysis score plot based on all silage metabolite components. (c) Variable importance in projection score of the metabolite compounds. Treatments included R0 = The control, R1 = Inoculation with Lactiplantibacillus *plantarum*, R2 = Supplementation with acacia leaf extract, and R3 = Combined application of both additives.

PCA and PLS-DA were performed to visualize the variations in metabolite profiles among the treatments (Figures 3a and b). Both score plots revealed clear separation among the *Indigofera* silage samples treated with different feed additives. In the PCA model, PC1 and PC2 explained 37.50% and 28.90% of the total variance, respectively, whereas in the PLS-DA model, Components 1 and 2 accounted for 37.40% and 23.30% of the variance, respectively.

These clustering patterns reveal pronounced differences in metabolite composition and concentration across treatments, underscoring the significant impact of feed additives on the fermentation-derived metabolite profile. VIP analysis was conducted to identify the metabolites contributing to the separation of the groups in the PLS-DA model. A total of 23 volatile metabolites with VIP >1 were detected in the *Indigofera* silage (Figure 3c), indicating their key role in differentiating silage treatments. Among these, propyl propanoate, 2-nonanol, 2-pentylbutanoate, 2-tetradecanone, and 2-methylundecanal showed the highest VIP scores.

#### Metabolic interpretation of key compounds

Propyl propanoate is an ester formed by the condensation of propanol and propanoic acid. Its presence in the volatile compound profile of silage is associated with the esterification reaction between alcohols and acids, a common process during ensiling. Isopropyl propanoate can be formed via the esterification of propanoic acid and 1-propanol [72]. The acid-to-alcohol ratio, fermentation temperature, acidic conditions, and the activity of microbial enzymes that act as catalysts in the silage system influence the rate and yield of this reaction.

2-Pentyl butanoate belongs to the ester group, whereas 2-nonanol belongs to the alcohol group. These findings are consistent with those of reported by Jiajie *et al*. [73], which show increased ester and alcohol contents during silage fermentation. Yeasts and other microorganisms produce high concentrations of alcohols, which can subsequently react with organic acids to form aromatic esters [74]. This reaction enhances the sweet aroma in silage. Furthermore, fermentation generates organic acids, such as acetic and butyric acids, which can undergo further conversion into esters, thereby enhancing the silage’s sweet flavor and aroma complexity [75].

The detection of propyl propanoate, 2-pentyl butanoate, and 2-nonanol indicated active microbial fermentation, supporting silage stability and contributing to the formation of desirable volatile profiles in *Indigofera* silage.

### Hierarchical clustering analysis (HCA) and compound differentiation

**Figure 4:**
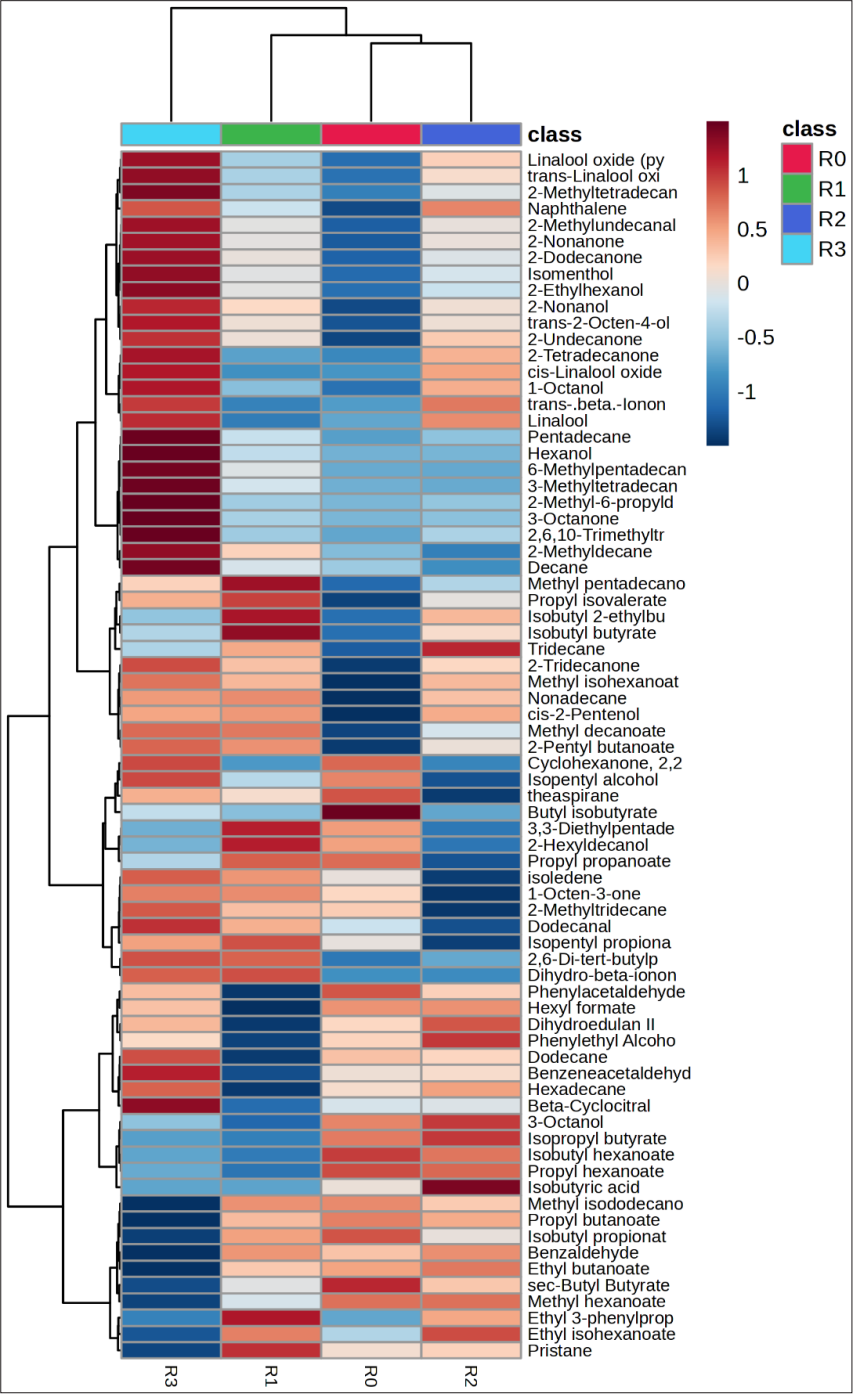
Hierarchical cluster analysis (heatmap) of silage metabolites from *Indigofera* colors represent the metabolite correlation levels with feed additives, ranging from +1 (red) to −1 (blue). Treatments included R0 = The control, R1 = Inoculation with *Lactiplantibacillus plantarum*, R2 = supplementation with acacia leaf extract, and R3 = Combined application of both additives.

HCA and the corresponding heatmap (Figure 4) revealed distinct differences in metabolite profiles between control and treatment groups. The dendrogram demonstrated proximity relationships among groups based on similarity in metabolite composition, showing that R0 clustered closely with R2, whereas R3 formed a separate branch with markedly different metabolic characteristics.

Compounds in the upper cluster, such as linalool oxide, 2-methylundecanal, and 2-nonanone, showed higher intensities in R3, whereas ester compounds, including methyl decanoate and 2-pentyl butanoate, were also more abundant in this treatment. In contrast, aromatic compounds such as benzaldehyde were more prominent in R1 and R2.

### Functional implications of additive synergy on metabolic pathways

The combination of *L. plantarum* and *Acacia* leaf extract increased the levels of linalool oxide, 2-methylundecanal, and 2-nonane, indicating enhanced terpenoid and secondary alcohol metabolism. This finding aligns with Weiss [76], who identified terpenoids as the major volatile compounds formed during ensiling. Ester compounds resulting from the microbial decomposition of OM contribute to silage’s characteristic sweet aroma [77].

In addition, the elevated levels of aromatic compounds, such as benzene, in the *Acacia*-extract treatment suggest activation of plant aromatic-degradation pathways through which microbes convert complex molecules into aldehydes and organic acids that influence silage aroma and flavor [78].

Metabolomic profiling offers valuable insights into these biochemical transformations and links microbial metabolism to silage’s fermentative, nutritional, and sensory properties [79]. The increased production of volatile compounds and activation of metabolic pathways not only enhance sensory quality but may also improve silage stability, nutrient utilization, and ruminant production performance.

### Untargeted metabolome profiles of the rumen fluid

#### Overall compound identification and classification

**Figure 5:**
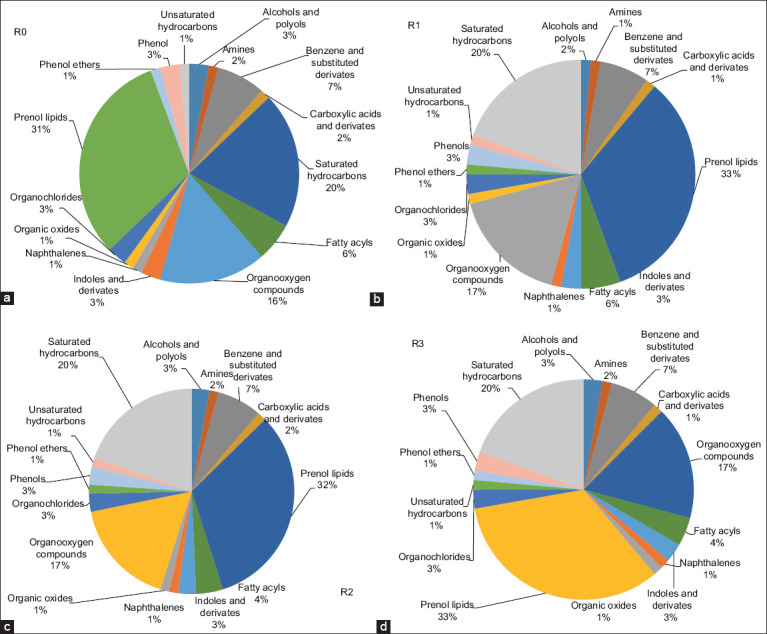
Class-based variation of metabolite compounds in *Indigofera* silage as classified by the Human Metabolome Database. Treatments included (a) R0 = The control; (b) R1 = Lactiplantibacillus *plantarum* inoculation; (c) R2 = *Acacia* leaf extract supplementation; (d) R3 = Combined application of both additives.

A total of 73 compounds were successfully identified in the rumen fluid based on their retention indices, retention times, and matching with the NIST 20 library. According to the HMDB database, these compounds were classified into several metabolite classes (Figure 5), including prenol lipids, phenols, indoles and their derivatives, and saturated hydrocarbons. Prenol lipids were the dominant group, accounting for 32%–33% in R1, R2, and R3, compared with 31% in the control (R0). Saturated hydrocarbons (18%–19%) and organooxygen compounds (16%–17%) were consistently detected across all treatments, with a slight reduction in hydrocarbon levels and a modest increase in organooxygen compounds observed in R2 and R3.

### Functional significance of rumen-derived metabolites

Rumen-derived metabolites play a crucial role in maintaining ruminant physiological balance and health. These compounds contribute to rumen homeostasis, modulate microbial fermentation, and influence metabolic efficiency and nutrient use [80]. The observed increase in prenol lipids is consistent with previous findings of Adawiah *et al*. [81], which show that feed additives can enhance terpenoid formation, underscoring their potential benefits in ruminant feed systems.

Prenol lipids, including sesquiterpenoids and monoterpenoids, are synthesized in the rumen through microbial biosynthetic pathways. The diversification of these terpenoids supports rumen function and microbial populations, and their antimicrobial and pharmacological activities can modulate fermentation dynamics and promote rumen health [82–84].

### Multivariate analysis of rumen metabolome

#### PCA and PLS-DA models

**Figure 6:**
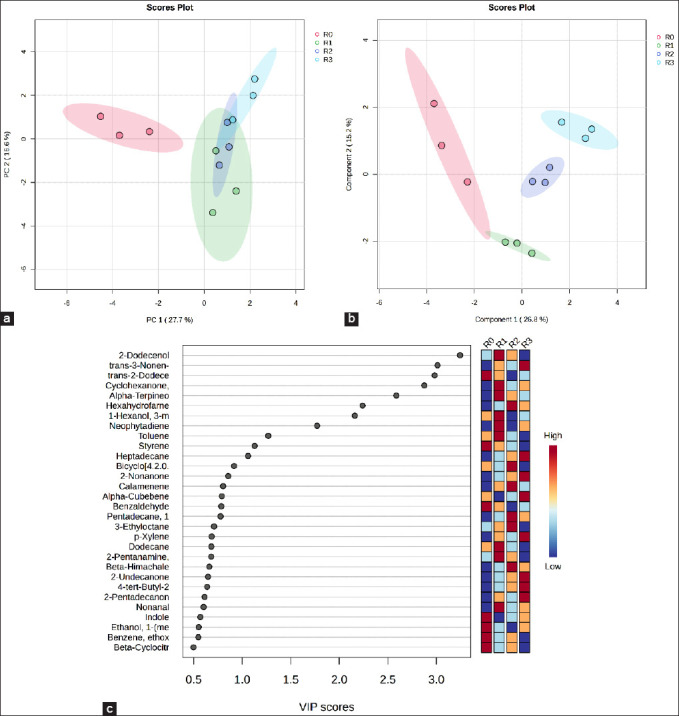
Metabolite profiles of rumen fluid with different feed additives. (a) Proportional coefficient analysis score plot illustrating variation among treatments. (b) Partial least squares discriminant analysis score plot based on all metabolite components. (c) variable importance in projection values of metabolite compounds. Treatments included R0 = the control, R1 = Inoculation with Lactiplantibacillus *plantarum*, R2 = Supplementation with acacia leaf extract, and R3 = Combined application of both additives.

PCA and PLS-DA were performed to assess variations in rumen metabolites among treatments (Figures 6a and b). The PCA score plot revealed a clear separation along PC1 (27.70%) and PC2 (16.60%), whereas the PLS-DA model showed distinct clustering along components 1 (26.80%) and 2 (15.20%). These results indicate marked differences in metabolite composition and concentration as influenced by feed additive treatments.

The VIP scores from PLS-DA identified 11 volatile compounds (VIP > 1) as major contributors to group discrimination (Figure 6c). The five metabolites with the highest contributions were 2-dodecanol, trans-3-nonen-2-one, trans-2-dodecen-1-ol, cyclohexanone, and α-terpineol.

#### Biochemical role of key rumen metabolites

2-Dodecanol is a reduction product of long-chain ketones formed during fatty acid degradation via fermentative microbial activity and may originate from lipid oxidation in *Indigofera* silage. The presence of *L. plantarum* can influence microbes involved in lipid metabolism by altering pH and substrate availability. The identification of trans-2-dodecen-1-ol is consistent with previous findings of Jiajie *et al*. [73], which show that ketone and phenolic metabolites are positively correlated with *Enterobacter, Escherichia–Shigella*, and *Clostridium*, but negatively correlated with unsaturated alcohols, such as trans-2-dodecen-1-ol.

These results highlight the significance of rumen microbial dynamics in the formation and transformation of long-chain alcohols. Moreover, ketone compounds exhibit antioxidant properties that may alleviate rumen oxidative stress and support livestock health and performance [85].

### Cluster relationship and functional implications

**Figure 7:**
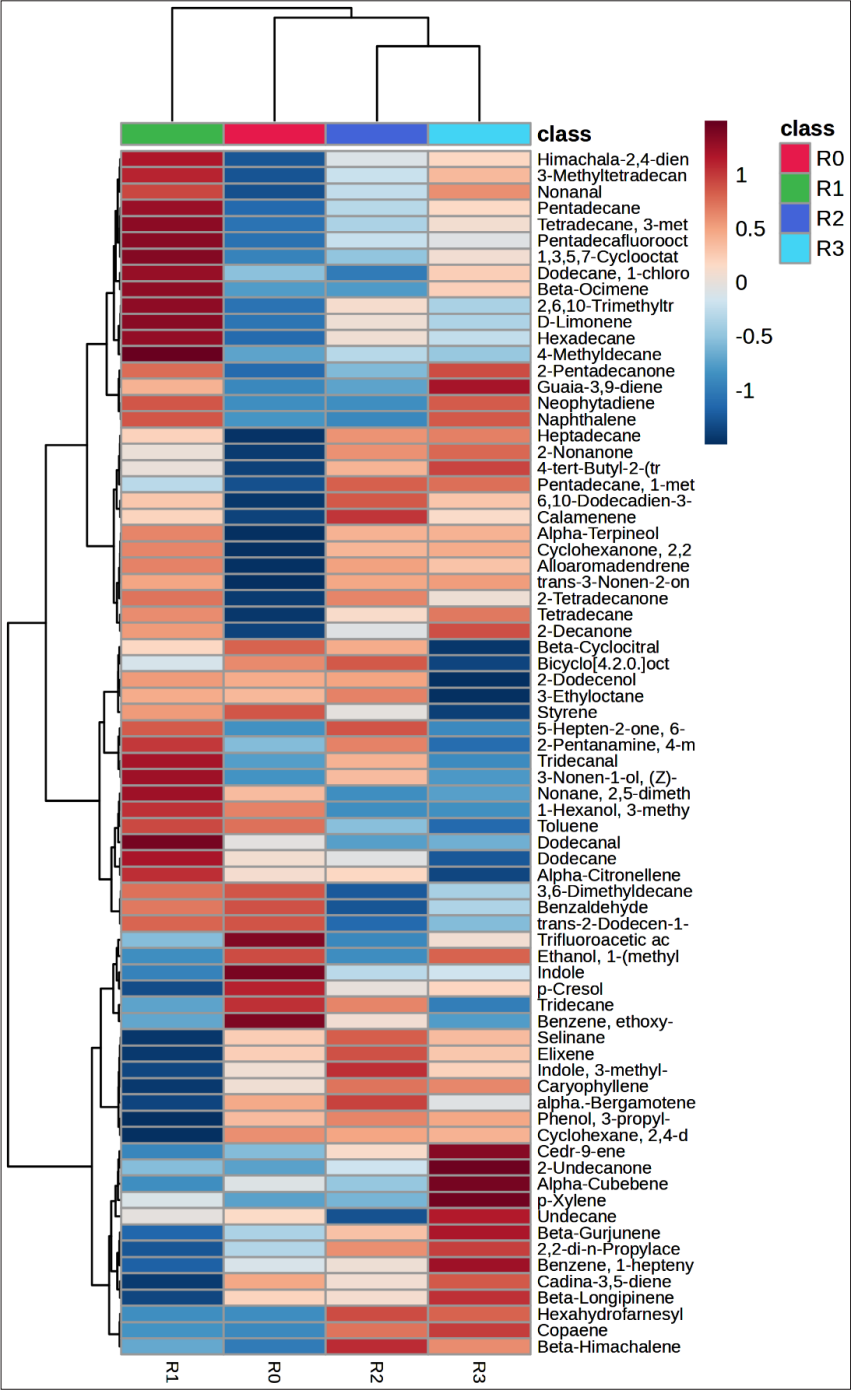
Hierarchical cluster analysis (heatmap) of metabolites in rumen fluid Colors represent the metabolite correlation levels with feed additives, ranging from +1 (red) to −1 (blue). Treatments included R0 = The control, R1 = Inoculation with Lactiplantibacillus *plantarum*, R2 = Supplementation with acacia leaf extract, and R3 = Combined application of both additives.

Figure 7 illustrates the relationships among the treatment groups based on their metabolite profiles. Group R2 exhibited the greatest similarity to R3, whereas R1 formed a distinct branch, and R0 clustered more closely with R2. This clustering pattern suggested that the combination of *L. plantarum* inoculum and *Acacia*-leaf extract (R3) generated a distinct yet related metabolic profile to the single *Acacia* treatment (R2).

The phenolic compound p-cresol showed the highest concentration in R0 and gradually decreased across R1, R2, and R3. Similarly, the control group (R0) contained high levels of indole, trifluoroacetic acid, benzaldehyde, benzene ethoxy-, and tridecane, which decreased with the addition of feed additives. In contrast, the indole derivative indole-3-methyl displayed a different trend, with elevated concentrations in R0, R2, and R3 but a marked reduction in R1. The terpenoid compound caryophyllene was detected at relatively high levels in both R2 and R3.

### Functional roles of indole and caryophyllene

Indole functions as a bacterial signaling molecule that mediates inter- and intra-species communication and regulates virulence, antibiotic resistance, and host immune interactions-associated genes [86]. It is synthesized in the rumen through the deamination and decarboxylation of tryptophan [87]. Elevated indole concentrations may adversely affect livestock products by contributing to undesirable odors in meat and milk. The reduction in indole formation following the addition of *Acacia* leaf extract is attributed to tannins, which inhibit microbial proteolysis and deamination, limiting indole formation [61]. This reduction has beneficial implications for rumen fermentation, as it indicates controlled protein degradation, improved nitrogen utilization, and enhanced microbial protein synthesis.

Caryophyllene, a sesquiterpenoid compound, increased following the addition of *Acacia* leaf extract, presumably because of its tannin content [88]. Microorganisms such as Enterobacter, Klebsiella, and Pseudomonas participate in the biodegradation of caryophyllene in the rumen [89]. This compound exhibits antioxidant and anti-inflammatory properties that help stabilize rumen microbial populations, maintain redox balance, and reduce the formation of toxic oxidative compounds [90]. Furthermore, its negative correlation with methanogenic archaea suggests that higher caryophyllene concentrations may suppress methanogenic activity and, consequently, lower CH_4_ production during rumen fermentation [91].

By promoting more stable rumen conditions, caryophyllene could enhance fermentation efficiency and nutrient use.

## CONCLUSION

The present study demonstrated that the application of *L. plantarum* inoculant, *A. mangium* leaf-extract tannins, and their combination significantly influenced the nutrient composition, fermentation quality, and rumen fermentation profile of *I. zollingeriana* silage. Among the treatments, the combined additive (R3) yielded the most desirable outcomes, characterized by a higher crude-protein content (up to 30.31% DM), reduced neutral- and acid-detergent fiber fractions, lower acid-detergent-insoluble protein, and enhanced lactic acid production, accompanied by a lower pH of approximately 4.6. The presence of tannins effectively reduced proteolysis and NH_3_-N accumulation, while *L. plantarum* accelerated acidification and improved fermentation efficiency. *In vitro* rumen evaluation further confirmed that the combined treatment improved dry- and organic-matter digestibility, enhanced propionate proportion, and reduced the C_2_:C_3_ ratio, indicating more energy-efficient fermentation and a potential reduction in enteric CH_4_ emission.

Untargeted metabolomic profiling using GC–MS revealed distinct differences in volatile compound spectra among treatments. In silage, ester and alcohol derivatives such as propyl propanoate, 2-nonanol, and 2-pentyl butanoate dominated, contributing to a desirable aroma and improved silage stability. In the rumen fluid, lipid-derived metabolites and terpenoids, such as caryophyllene and α-terpineol, were prominent in the *Acacia*-treated groups, suggesting antioxidant and anti-methanogenic potential. The metabolomic evidence supports a biochemical synergy between *L. plantarum* and plant tannins, enhancing fermentation dynamics and nitrogen retention.

These findings highlight a practical and sustainable approach for tropical livestock systems, where *A. mangium* leaves, a low-cost forestry by-product, can serve as a natural tannin source to enhance the preservation and nutritive value of *Indigofera* silage. The combination with a proven LAB inoculant offers a biological strategy to reduce nitrogen losses, improve protein efficiency, and potentially mitigate CH_4_ emissions, thereby aligning feed management with circular and climate-smart agriculture.

The major strength of this study lies in its integrative design, which links silage quality, *in vitro* rumen fermentation, and metabolomic profiling within a single experimental framework. However, the research was conducted under *in vitro* conditions, which do not fully replicate animal feeding environments, and the relatively low tannin concentration, along with the absence of *in vivo* performance data, limit the direct extrapolation of productivity or CH_4_-reduction effects.

Future studies should validate these results *in vivo* using different tannin inclusion rates, purified tannin fractions, and long-term feeding trials to assess animal performance, nitrogen balance, and emission profiles. Integrating microbial sequencing with metabolomics would further elucidate microbial pathways underlying tannin–LAB interactions.

In conclusion, co-application of *L. plantarum* and *A. mangium* tannins effectively improved the nutritional quality, fermentation stability, and metabolic profile of *Indigofera* silage. This synergistic, eco-friendly technology represents a promising strategy for enhancing forage utilization efficiency and promoting sustainable ruminant production in tropical regions.

## DATA AVAILABILITY

All the generated data are included in the manuscript.

## AUTHORS’ CONTRIBUTIONS

FRA: Performed the entire experiment, collected the data, and drafted the manuscript. IS collected data and drafted the manuscript. RoR, RF, and YW: Performed the *in vitro* experiments. NN, YRY, VN, and RR: Supervised the study, checked the data analysis, and revised the manuscript. AJ designed and supervised the study, checked the data analysis, and revised the manuscript. All authors have read and approved the final version of the manuscript.
